# Cardiology Provider Knowledge and Attitudes Toward Cardiac Gene Therapy: A Multi-Institutional National Survey

**DOI:** 10.1016/j.jchf.2026.103065

**Published:** 2026-04-24

**Authors:** Elizabeth Silver, Megan Punt, Jessica Wang, Alison Brann, Rola Khedraki, Salil Kumar, Antonio Duran, Barry Greenberg, Kimberly N. Hong, Quan M. Bui

**Affiliations:** a Division of Cardiovascular Medicine, Department of Medicine, University of California-San Diego, La Jolla, California, USA; b Department of Medicine, Massachusetts General Hospital, Boston, Massachusetts, USA; c Division of Cardiology, Department of Medicine, School of Medicine, University of Colorado, Aurora, Colorado, USA; d Division of Cardiology, Department of Medicine, University of California-Los Angeles, Los Angeles, California, USA; e Division of Cardiology, Department of Medicine, University of Utah, Salt Lake City, Utah, USA; f Division of Cardiology, Department of Medicine, Scripps Health, La Jolla, California, USA; g Department of Cardiology, Division of Internal Medicine, MD Anderson Cancer Center, Houston, Texas, USA; h Division of Cardiology, Department of Medicine, Sharp Health, San Diego, California, USA; i Altman Clinical and Translational Research Institute Dissemination and Implementation Science Center, University of California-San Diego, La Jolla, California, USA

**Keywords:** cardiovascular genetics, gene therapy, implementation, provider attitudes

As gene therapy (GT) advances toward clinical implementation in cardiovascular disease, clinician preparedness will be critical. Cardiovascular GT programs are progressing through early-phase trials, yet these therapies carry substantial risks, including immunologic toxicity, uncertain durability, single-dose limitations, and high cost.^1,2^ Successful integration into practice will require cardiologists to counsel patients, identify eligible candidates, and navigate referral pathways. However, data on cardiology provider readiness remain limited. We conducted a multi-institutional survey to assess knowledge, attitudes, perceived barriers, and educational priorities among cardiology providers regarding GT.

## METHODS

Survey items were developed based on the review of published reports and author expertise and iteratively refined through review by independent cardiologists and implementation scientists to ensure content relevance and clarity. The 25-item electronic survey was distributed once via institutional contacts to cardiology attendings, fellows, and advanced practice providers (APPs) at 7 academic and community centers and remained open between June and September 2025. The items included multiple-choice, Likert scale, and multiple-selection response formats. The responses were summarized using descriptive statistics. Knowledge scores were calculated as the number of correct true/false responses and stratified as low (<7 of 10) or high (≥7 of 10). The study was institutional review board-approved with exempt status.

Of 605 providers contacted, 123 complete surveys were included (response rate 20.3%). The respondents included 49 attending physicians (40%), 32 fellows (26%), and 42 APPs (34%). Thirty-five percent had been in practice <5 years and 17% >20 years. Most practiced in academic settings (94%). Common subspecialties included general cardiology (46%) and advanced heart failure/transplant (30%).

## RESULTS

**FIGURE 1:**
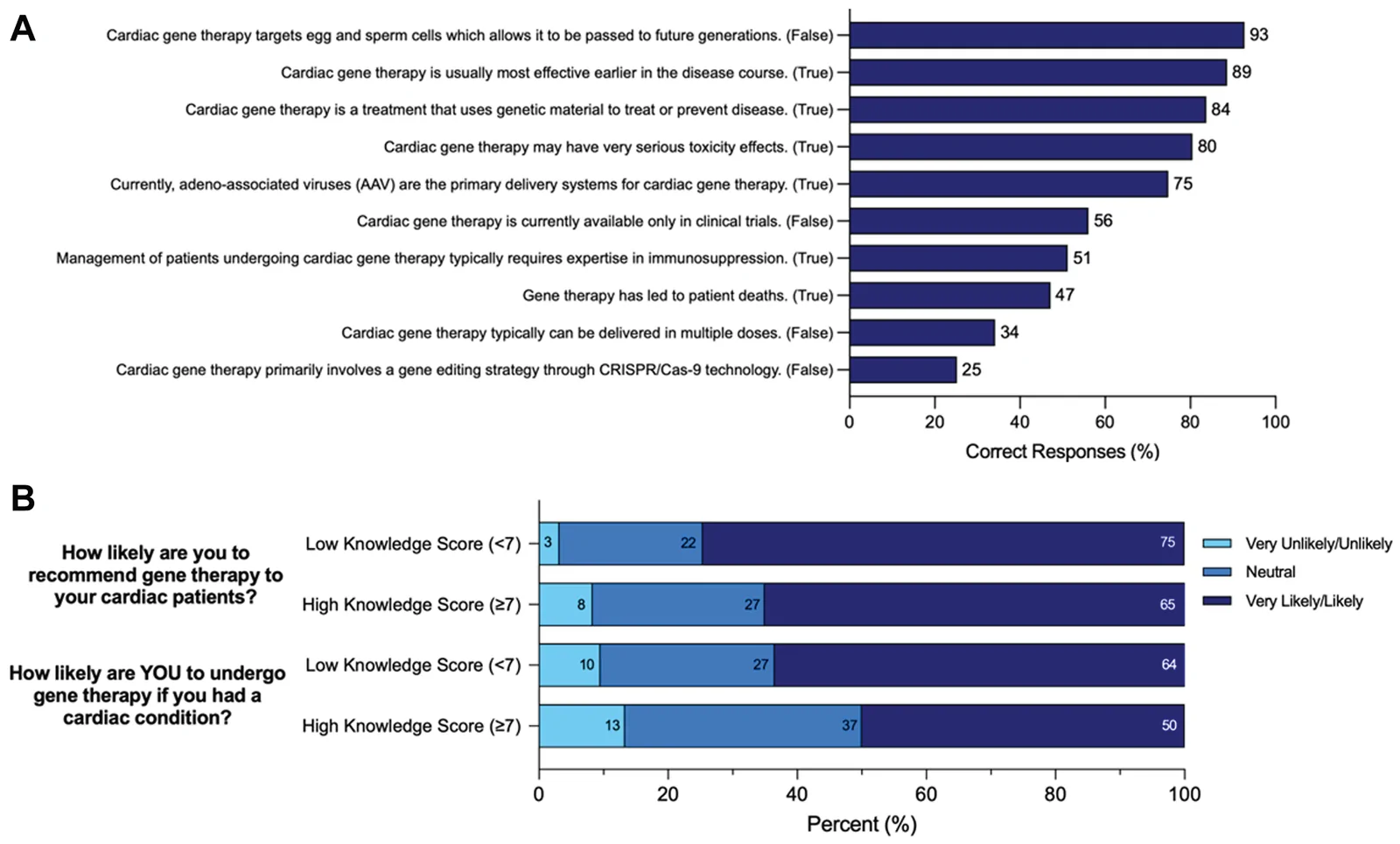
Knowledge and Willingness to Undergo GT (A) Percent correct responses for knowledge-based true/false questions. (B) Likelihood of recommending gene therapy to cardiac patients and personally undergoing gene therapy, stratified by knowledge score (low <7 correct responses, n = 63; high ≥7 correct responses, n = 60). Bars represent the proportion of respondents reporting very unlikely/unlikely, neutral, or very likely/likely responses. GT = gene therapy.

Self-rated familiarity with cardiac GT was modest: although 79% reported being at least somewhat familiar with basic principles, <20% rated their understanding of current research as good or excellent. Nearly 75% reported they were not familiar with the clinical trial enrollment processes. On objective knowledge items (Figure 1A), most recognized that GT is not heritable (93%), is more effective earlier in disease (89%), uses genetic material to treat disease (84%), and may cause toxicity (81%). However, fewer participants correctly identified that most current cardiac GTs do not use CRISPR (Clustered regularly interspaced palindromic repeats)/Cas9 editing (25%), recognized that therapies are typically delivered as a single dose (34%), were aware that GT has resulted in patient deaths (47%), and recognized the need for immunosuppression expertise (51%).

Despite knowledge gaps, enthusiasm was high. Most participants agreed that cardiac GT has the potential to improve patient outcomes (95%) and were likely to recommend GT to eligible patients (70%). However, the participants were hesitant to undergo GT themselves if they had a cardiac condition (43%). When presented with a scenario involving a highly morbid condition without effective alternatives, willingness increased for both recommending and personally undergoing GT (85%).

Providers identified lack of knowledge or training (67%), high cost (49%), and long-term safety concerns (45%) as major barriers. Education (80%), access to genetic counseling (56%), and institutional support (53%) were the most commonly cited facilitators. Institutional awareness was limited: fewer than half (47%) reported that their institution had an inherited cardiovascular disease clinic (although such clinics existed at 6 of 7 sites), and 50% were unsure whether their institution was an active GT trial site. Most providers (85%) had never referred a patient to a GT clinical trial.

Training in genetics and genomics was limited, with only 30% reporting moderate or extensive exposure. Sixty-two percent expressed interest in learning more about GT, preferably through clinical guidelines (53%), in-person workshops (50%), and webinars or videos (50%). Highly requested learning topics included safety considerations (69%) and available clinical trials (60%).

In descriptive subgroup analyses, the respondents with higher knowledge scores (n = 60) were less willing to recommend GT (65% vs 75%) and less willing to personally undergo GT (50% vs 64%) compared with those with lower knowledge scores (n = 63) (Figure 1B). Attending physicians and advanced heart failure/transplant specialists demonstrated slightly higher objective knowledge accuracy than fellows/APPs and general cardiologists, respectively, but reported a lower personal willingness to undergo GT.

## DISCUSSION

To our knowledge, this is the first multi-institutional survey of U.S. cardiology providers evaluating preparedness for cardiac GT. A clear readiness gap emerged: although nearly all respondents believed GT has the potential to improve outcomes (95%), their objective knowledge, familiarity with trial processes, and awareness of institutional resources were limited. Only 24% reported moderate or high familiarity with GT fundamentals, and 85% had never referred a patient to a GT trial. Many were unaware of existing inherited cardiovascular disease clinics or active GT trials at their own institutions. Together, these findings suggest that enthusiasm may currently outpace implementation preparedness in cardiovascular practice, similar to previously described gaps in provider confidence regarding cardiogenetic testing.^3^

The knowledge gaps were not uniform. Attending physicians and advanced heart failure/transplant specialists demonstrated greater familiarity and objective knowledge than fellows/APPs and general cardiologists, suggesting that exposure to inherited cardiomyopathies, immunosuppression, and genetics may shape preparedness. As GT enters later-phase trials, heart failure/transplant programs may serve as early hubs of implementation; however, broader education across general cardiology and advanced practice providers will be necessary to ensure timely identification and referral of eligible patients.

Notably, greater knowledge was associated with lower willingness to personally undergo GT. This paradox, which has also been observed with other high-risk cardiovascular therapies such as left ventricular assist device implantation,^4^ may reflect a heightened awareness of toxicity, uncertainty, and logistical complexity among more informed clinicians. Rather than representing resistance, this pattern may signal a more calibrated risk perception. As cardiac GT advances, clinician education must balance optimism with transparent discussion of safety, durability, and cost to support informed counseling and shared decision-making.

Beyond individual knowledge deficits, system-level barriers were prominent. Limited awareness of institutional genetics resources and active clinical trials suggests that implementation challenges may stem as much from communication and infrastructure gaps as from training deficits. Improving the visibility of inherited cardiovascular disease programs, integrating genetic counseling within heart failure care pathways, and standardizing referral processes may be as critical as didactic education.

### STUDY LIMITATIONS.

This study has limitations, including reliance on self-report, predominance of academic centers, and descriptive analyses without formal instrument validation. Nonetheless, these data provide early insight into clinician readiness as cardiac GT approaches broader clinical translation.

## CONCLUSIONS

Cardiology providers express strong optimism regarding cardiac GT, yet meaningful knowledge and systems gaps persist. Addressing both individual and institutional readiness will be essential to ensure safe, equitable adoption as gene-based therapies enter cardiovascular practice.

## What is the clinical question being addressed?

What are cardiology provider attitudes and readiness for cardiac gene therapy?

## What is the main finding?

Cardiology providers showed high enthusiasm for cardiac gene therapy but had limited knowledge and awareness of institutional resources as well as clinical trials, highlighting implementation opportunities.

## ABBREVIATIONS AND ACRONYMS

APP: advanced practice provider
GT: gene therapy
